# Riluzole is associated with reduced risk of heart failure

**DOI:** 10.1111/ene.70033

**Published:** 2025-01-09

**Authors:** Kibum Kim, Sodam Kim, Margaret Katana, Dmitry Terentyev, Przemysław B. Radwański, Mark A. Munger

**Affiliations:** ^1^ Department of Pharmacy Systems and Outcomes and Policy University of Illinois Chicago Chicago Illinois USA; ^2^ Department of Physiology and Cell Biology The Ohio State University Columbus Ohio USA; ^3^ The Frick Center for Heart Failure and Arrhythmia Dorothy M. Davis Heart and Lung Research Institute, College of Medicine, The Ohio State University Wexner Medical Center Columbus Ohio USA; ^4^ Division of Pharmaceutics and Pharmacology College of Pharmacy, the Ohio State University Columbus Ohio USA; ^5^ Department of Pharmacotherapy University of Utah Health Salt Lake City Utah USA; ^6^ Department of Internal Medicine University of Utah Health Salt Lake City Utah USA

**Keywords:** heart failure, Riluzole, small conductance Ca^2+^‐activated K^+^ channels, sodium channels

## Abstract

**Background:**

Reduction of intracellular Na^+^ accumulation through late Na^+^ current inhibition has been recognized as a target for cardiac Ca^2+^ handling which underlies myocardial contractility and relaxation in heart failure (HF). Riluzole, an Na^+^ channel blocker with enhancement of Ca^2+^‐activated K^+^ channel function, used for management of amyotrophic lateral sclerosis (ALS), is effective in suppressing Ca^2+^ leak and therefore may improve cardiac function.

**Objectives:**

The study aim was to investigate whether riluzole lowers HF incidence.

**Methods:**

Rates of HF incident were compared using a commercial insurance and Medicare supplement claims databases. Patients with a filled riluzole prescription (treatment) between 06/2009 and 12/2019 were compared to those with no‐riluzole (control). We excluded HF patients during the 180‐day baseline period. Study endpoint was the first HF diagnosis from the index riluzole prescription or ALS diagnosis. HF onset was compared between the propensity score matched treatment and control cohorts.

**Results:**

The matched cohort consisted of 4060 pairs of riluzole/control patients. The 24‐month cumulative incidence of HF onset for riluzole versus control patients was 4.96% versus 7.27%, calculating hazard ratio (HR) [95% CI, *p*‐value] of 0.55 [0.40–0.76, *p* < 0.01]. The HR estimates favoring riluzole over the ALS control were consistent across the 3 months to 2‐year follow‐up. The clinically and statistically significant effect on HF onset was driven by the lower rate of HFrEF with the 2‐year HR [95% CI] of 0.46 [0.21–0.99].

**Conclusions:**

Riluzole is associated with a lower rate of HF onset, suggesting a potential prevention strategy for early management.

## INTRODUCTION

Heart failure (HF), a progressive continuum of ventricular dysfunction, remains a major unsolved growing health and economic burden, despite therapeutic advances [1, 2]. Through the cardiovascular continuum, there are attributable risk factors that elevate the risk for HF development which include hypertension, prediabetes, diabetes, atherosclerotic cardiovascular disease, and non‐ischemic lesions [3, 4]. HF risk progresses with the development of clinically asymptomatic structural and functional cardiac abnormalities. Estimates of patients ≥45 years of age, with risk factors or who have asymptomatic ventricular dysfunction, is 56% of the U.S. population [4]. Hence, there is an urgent need to identify new therapeutic strategies to address this unmet cardiac health problem.

Targeting intracellular Na^+^ accumulation in HF has been recognized as a potential strategy in alleviating symptoms associated with HF [5, 6, 7, 8]. Increasing Na^+^ levels lead to intracellular Ca^2+^overload through the Na^+^/Ca^2+^ exchange and mitochondrial dysfunction, which in turn impacts cardiac contraction and relaxation [9, 10].

Cardiac small conductance Ca^2+^‐activated K^+^ (SK) channels are thought to play important roles in cardiac and vascular protection as well [11]. Sarcolemmal SK channels reduce pro‐arrhythmic disturbances in membrane potential in Ca^2+^‐overloaded myocytes [12]. In addition, mitochondria‐residing SK channels were shown to reduce mitochondrial dysfunction in ischemia–reperfusion injury and pressure overload‐induced hypertrophy [13, 14], improving aberrant intracellular Ca^2+^ homeostasis.

Riluzole is used to manage amyotrophic lateral sclerosis (ALS) [15] with a dual mechanism of promoting Na_V_ blockade as well as enhancing SK channel function [16, 17, 18, 19]. It has proven effective in suppressing Ca^2+^ release in multiple models [20, 21, 22, 23]. Based on these findings, we hypothesized that riluzole would reduce the incidence of HF in patients with American College of Cardiology (ACC)/American Heart Association (AHA) Stage A or B heart failure [3]. Therefore, the purpose of this investigation was to test whether riluzole in real‐world data science could mitigate the risk of developing HF.

## METHODS

### Study design and population

A retrospective cohort study using an insurance claims database was performed. Healthcare encounters and outpatient pharmacy records were obtained from the Truven Health MarketScan® Commercial Claims and Medicare Supplemental databases (Truven Health Analytics, Ann Arbor, MI), which covered medical claims of commercially insured patients from January 2009 to December 2019. The database does not include patient‐level identifiable information and did not qualify for human subject research. This study was deemed exempt by the University of Illinois Institutional Review Board.

Riluzole dispensing was the exposure of interest with or without the presence of ALS diagnosis, defined by the National Drug Codes available from the outpatient pharmacy service records (Supplement). The control cohort included patients with one or more ALS diagnosis medical encounters (Table S1). The first date of ALS diagnosis in patients without riluzole was labeled as an index date. The analytic cohort included male or female patients with the index exposure or ALS. We excluded records <18 years old at the index date. To address baseline characteristics, all patients remaining in the analysis ≥180 days from the baseline enrollment period before the index date with <1 month coverage gap.

We used two analytic cohorts for this study. First, an exploratory analysis cohort included all the patients meeting aforementioned criteria. A subset of the exploratory analysis cohort after excluding patients with any records of HF during the 180‐day baseline period became the primary outcome cohort for an initial analysis. Second, a matched analytic cohort was created from and nested within the primary outcome cohort, which was the final analytic cohort.

### Outcomes

The primary outcome was the onset of HF (HF Onset) after index date among patients without baseline HF records. HF onset was defined by ICD‐9 or ICD‐10 CM of HF at any position from an outpatient or inpatient service encounter. Analysis of HF onset was performed using the primary outcome and matched analytic cohorts. The use of data for this study was reviewed and determined to be exempt from human subjects' research by the Institutional Review Board of the University of Illinois at Chicago.

We analyzed HF admission (HF ADM) as an exploratory outcome. HF ADM was defined by a primary, secondary, or tertiary HF diagnosis from an admission summary claim. All patients included in the exploratory cohort were subjects included in the HF ADM analysis.

### Statistical analysis

Descriptive statistics and bivariate analyses were used to compare baseline characteristics between the exposure and control cohort. Age distributions were compared using mean and standard deviation (SD) and a Student's *t*‐test. Categorical variables including grouped age (≤ 45, 45–64.9, ≥65), gender, region, type of health plan (commercial insurance general vs. Medicare supplemental), and baseline comorbid conditions included in the Charlson Comorbidity Index were compared with chi‐squared test. A Fisher exact test was used instead of a chi‐squared test when the expected number of patients in a cell of a frequency table was fewer than 5.

We compared the outcomes between the exposure and control cohorts using time‐to‐event analysis. The analysis was performed within the intention‐to‐treat frame, meaning that patients were followed from the index through the event or disenrollment regardless of the treatment continuation. We calculated Kaplan–Meier cumulative incidence. Patient records were censored with the disenrollment from the database, an enrollment gap ≥30 days to ≤2‐year (730‐day period) maximum follow‐up period or outcome of interest, whichever happened first. The hazard ratio (HR) of HF ADM or HF onset for the riluzole exposure was calculated using a Cox proportional hazard regression model for which baseline characteristics with *p* < 0.05 were adjusted.

### Matched cohort analysis

Propensity score matching (PSM) was performed to address the effect of observable confounders in the exposure—HF onset association. The odds of being assigned to the riluzole exposure were calculated using a logistic regression model where covariates entered and stayed in the model with *p* < 0.15 and stay *p* < 0.1 criteria, respectively. The final model included the following dependent variables: gender, insurance type, geographic region, dementia, myocardial Infarction, cerebrovascular accident, diabetes, liver disease, peripheral vascular disorders, semi/paraplegia, pulmonary disease, and renal disease. We used a 1:1 greedy matching algorithm within 0.2 times the pooled standard deviation (SD) of the logit of propensity score by selected covariates. Cumulative incidence and HR calculations for HF onset were performed using both matched and matched cohorts. Calculating HRs from the matched cohort, we adjusted for any residual confounders (*p* < 0.05) that were not fully addressed in the propensity score matching.

### Sensitivity analysis

The effect of riluzole on HF onset may vary by HF subtype, patient age, and confirmed ALS diagnosis. In order to test the influence of the cohort selection criteria and HF subtype, we ran the following sensitivity analyses. First, we replicated the analysis of HF onset for the two subtypes of the outcome, HF with preserved ejection fraction (HFpEF) and HF with reduced ejection fraction (HFrEF). Second, the matched cohort analysis was stratified by the age, ≥65 and <65 years old. To ensure that patients with riluzole and a baseline ALS diagnosis and those with riluzole, but without an ALS diagnosis, provide similar insights regarding the measure of association, we conducted a sensitivity analysis limiting the cohort to patients with a baseline ALS diagnosis.

ACEi and ARB demonstrated mortality benefits and may influence both onset of complication or censoring from the follow‐up. In part of the effort to address the confounding by the benefits of concurrent medications, we also performed the measure of effect calculation after excluding patients who were on ACEi or ARB during the baseline period.

## RESULTS

### Patient characteristics

**FIGURE 1:**
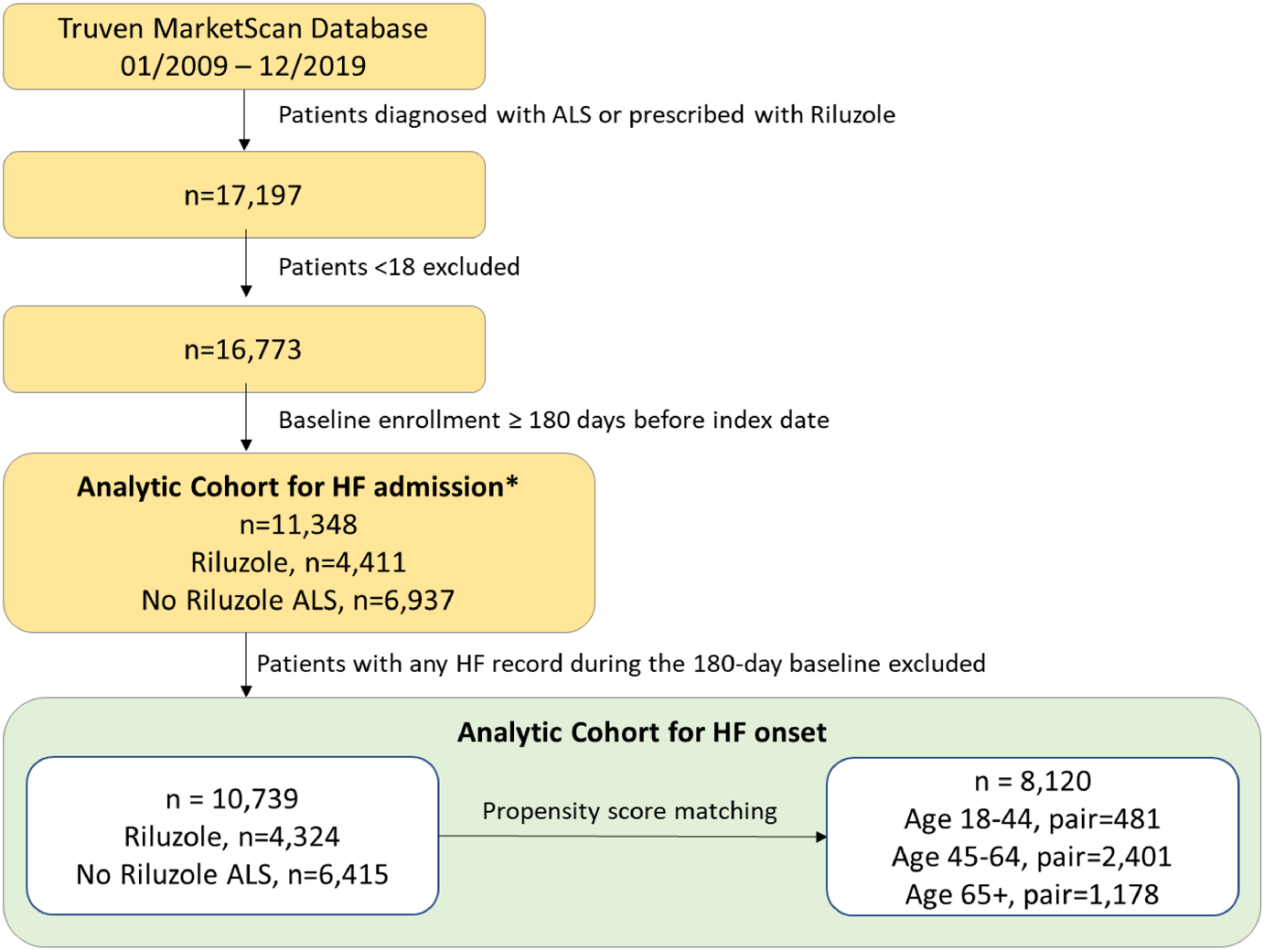
Cohort Extraction Flow Chart. *Analytic Cohort for HF admission: Patients may or may not have diagnoses of HF during the baseline period.

We identified 17,197 patients with ALS diagnosis or riluzole‐dispensing records. After applying patient selection criteria, the exploratory cohort included 11,378 unique patients consisting of 4441 riluzole and 6937 ALS controls (Figure 1). Riluzole patients were younger than the controls with respective age of (mean ± SD) of 59.08 ± 12.68 versus 62.31 ± 14.73. Control patients generally carried more comorbid conditions. Specifically, the prevalence of HF was significantly lower among the riluzole cohort (1.97%) than the controls (7.52%; Figure S1).

The primary outcome cohort included a total of 10,739 patients (4324 riluzole and 6415 ALS controls), after excluding patients with any baseline HF (Figure 1). Riluzole patients were younger than the ALS controls (58.92 ± 12.63 years old vs. 61.53 ± 14.48 years old) with 29.02% versus 39.17% of the respective cohorts 65 years or older. Although patients with baseline HF records were excluded, prevalence of a comorbidity that may influence the cardiovascular adverse events remained higher among the riluzole exposure cohorts compared to the ALS control (Table 1). Compared to the ALS controls, a significantly larger proportion of the riluzole patients received cardioprotective agents during the baseline periods, including angiotensin‐converting enzyme inhibitors (ACEi), angiotensin receptor blockers (ARB), beta blockers, Ca^2+^ channel blockers (CCB), metformin, and anti‐lipid agents (Table 1).

**TABLE 1:** Baseline characteristic for analytic cohorts for HF onset.

Characteristic	Unmatched (*N* = 10,739)			Matched (*N* = 8120)		
	Riluzole (*n* = 4324)	No riluzole (*n* = 6415)	*p*‐value	Riluzole (*n* = 4060)	No riluzole (*n* = 4060)	*p*‐value
	*n* (%)	*n* (%)		*n* (%)	*n* (%)	
*Demographic characteristics*						
Male	2401 (55.53)	3757 (58.57)	<0.01	2263 (55.74)	2243 (55.25)	0.66
Age (years, Mean ± SD)	58.92 ± 12.63	61.53 ± 14.48	<0.01	58.83 (12.76)	58.95 (13.35)	0.67
Age category, yrs.			<0.01			1.00
<45	496 (11.47)	686 (10.69)		481 (11.85)	481 (11.85)	
45–64	2573 (59.51)	3216 (50.13)		2401 (59.14)	2401 (59.14)	
≥65	1255 (29.02)	2513 (39.17)		1178 (29.01)	1178 (29.01)	
*Charlson's index score*						
Mean ± SD	2.25 (1.72)	2.70 (2.00)	<0.01	2.26 (1.74)	2.27 (1.77)	0.90
*Baseline comorbidity*						
Myocardial infarction	44 (1.02)	148 (2.31)	<0.01	44 (1.08)	36 (0.89)	0.37
Cerebrovascular Accident	700 (16.19)	1110 (17.30)	0.13	655 (16.13)	620 (15.27)	0.29
Diabetes	480 (11.10)	947 (14.76)	<0.01	462 (11.38)	437 (10.76)	0.38
Peripheral vascular disease	115 (2.66)	256 (3.99)	<0.01	113 (2.78)	106 (2.61)	0.63
Renal disease	39 (0.90)	38 (0.59)	0.06	34 (0.84)	22 (0.54)	0.11
*Baseline medication*						
ACEi	665 (15.38)	781 (12.17)	<0.01	622 (15.32)	567 (13.97)	0.08
ARB	378 (8.74)	353 (5.50)	<0.01	330 (8.13)	280 (6.90)	0.04
Beta blocker	771 (17.83)	855 (13.33)	<0.01	668 (16.45)	620 (15.27)	0.14
Calcium channel Blocker	572 (13.23)	645 (10.05)	<0.01	516 (12.71)	458 (11.28)	0.05**
Other CV drugs^a^	70 (1.62)	113 (1.76)	0.58	61 (1.50)	68 (1.67)	0.53
Insulin	84 (1.94)	151 (2.35)	0.15	83 (2.04)	82 (2.02)	0.94
Metformin	148 (3.42)	142 (2.21)	<0.01	134 (3.30)	113 (2.78)	0.17
SGLT2i	14 (0.32)	7 (0.11)	0.01	13 (0.32)	5 (0.12)	0.06
Other antidiabetic agents	129 (2.98)	183 (2.85)	0.69	120 (2.96)	112 (2.76)	0.59
Antiplatelet agents	173 (4.00)	221 (3.45)	0.13	152 (3.45)	138 (1.99)	0.40
Anti‐lipid agent	1231 (28.47)	1332 (20.76)	<0.01	1037 (23.51)	1038 (14.96)	0.98
Spironolactone	17 (0.39)	35 (0.55)	0.26	17 (0.39)	12 (0.17)	0.35

^a^ Other CV drugs includes alpha‐beta blockers, antiarrhythmic agents and cardiac glycosides.

** *p*‐value of 0.0476.

After propensity score matching, 8120 patients (4060 pairs) remained in the analysis. Demographics and clinical characteristics were well balanced between the riluzole exposure and ALS controls with similar age (riluzole vs. ALS control; 58.83 ± 12.76 vs. 58.95 ± 13.35), sex (55.74 vs. 55.25% male), geographic location (23.62% vs. 21.95% Eastern US). No concurrent conditions were significantly different at the index date (*p*‐values >0.11; Table 1). While the proportion of patients on β‐blockers, anti‐lipid agents, and metformin was not significantly different after matching, that for ACEi, ARB, CCB, and Na^+^‐glucose co‐transporter 2 inhibitors was either significantly or marginally different after matching. Those potential residual confounders were subsequently adjusted in a multivariable hazard regression model.

The aggregate population was relatively free from concomitant disease or disorders, except the presence of ALS, principally middle‐aged, 56%–44% male‐to‐female apportioned, and at‐risk for heart failure from the presence of risk factors and/or structural heart disease. Atherosclerotic disease was recorded in approximately 17% of the cohort noted by a history of cerebrovascular accident or myocardial infarction. Diabetes was recorded in 11%+ of the cohort.

### Association between riluzole exposure and HF incidence

**FIGURE 2:**
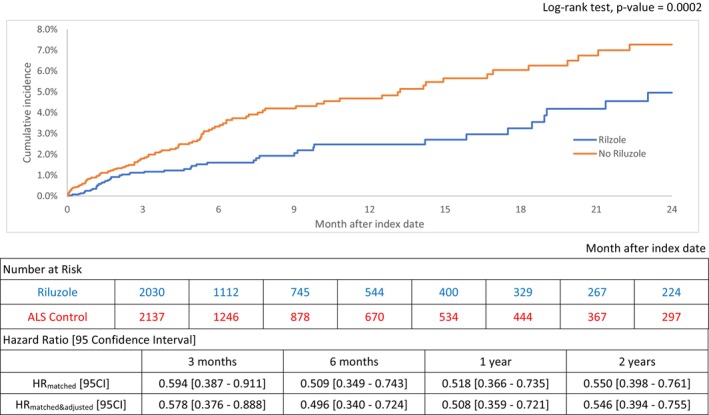
Two‐year cumulative incidence and hazard ratio estimates of HF onset for riluzole: Matched cohort.

The matched cohort analysis calculated a lower rate of HF incidence at 24 months after riluzole exposure versus ALS control with the respective cumulative incidence of 4.96% versus 7.27% (log‐rank test, *p* = 0.0002; (Figure 2). The hazard ratio (HR) and 95% confidence interval [95 CI] of HF incidence for riluzole versus control at 3‐, 6‐, 12‐, and 24‐month follow‐up were 0.578 [0.376–0.888], 0.496 [0.340–0.724], 0.508 [0.359–0.721], and 0.546 [0.394–0.755], respectively, after being adjusted for the residual confounders (Figure 2). In the unmatched cohort analysis, the riluzole group demonstrated a lower incidence rate of HF at 24 months, with the cumulative incidences of HF 4.71% in the riluzole group compared to 8.73% in the control group. Statistically significant HR estimates that favor riluzole in HF incidence were consistently observed from the analysis of the unmatched cohort. Adjusted HR [95 CI] at the respective follow‐up periods were 0.476 [0.323–0.703], 0.428 [0.302–0.607], 0.448 [0.324–0.619], 0.466 [0.345–0.630] in the unmatched cohort (Figure S1).

From the exploratory cohort, the risk of HF ADM was lower among the riluzole exposures than ALS controls with the 730‐day Kaplan–Meier cumulative incidence estimates of 0.33% versus 1.21 (log‐rank test, *p* = 0.0429; Figure S2). The lower cumulative incidence of HF ADM with riluzole calculated the 24‐month crude hazard ratio of 0.248 [0.105–0.583]. The measure of effect was statistically insignificant (hazard ratio of 0.411 [0.154 ‐ 1.096]) after adjusting for the baseline characteristics (Figure S2).

### Sensitivity analyses

**FIGURE 3:**
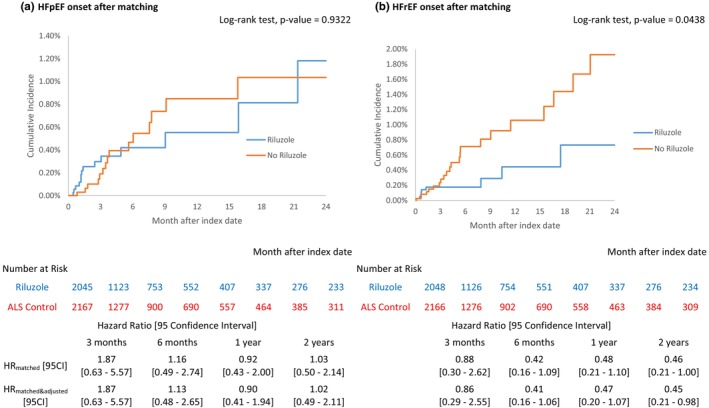
Two‐year cumulative incidence and hazard ratio estimates by HF subtype (HFpEF and HFrEF): Matched cohort analysis.

The measure of association was sensitive to HF subtypes. From the matched cohort analysis, 730‐day HR [95 CI] for HFpEF and HFrEF was 1.018 [0.491–2.113] and 0.452 [0.208–0.984], respectively (Figure 3). Similarly, non‐matched analysis resulted in the substantially larger and statistically significant association with HFrEF with HR [95 CI] of 0.386 [0.187–0.797] compared to the estimate for HFpEF of 0.769 [0.408–1.449] (Figure S3).

When the analysis was limited to the patients with ALS diagnosis at baseline, the 730‐day cumulative incidence for the riluzole exposures and controls was 5.07% and 8.73%, respectively, calculating the HR [95 CI] of 0.458 [0.331–0.633]. Excluding the riluzole patients without an observed ALS diagnosis did not influence the measure of association (Figure S4).

An association of HF incidence by age was also determined. From the matched cohort analysis of 730 days stratified by ≥65 years versus < 65 years of age HR was 0.79 [0.51–1.22] and 0.37 [0.22–0.60] (Figure S5).

ACEi or ARB has a modest impact on the association. After excluding those who were on ACEi or ARB during the baseline period, the HR [95 CI] approached toward null. However, the majority of the estimates were still less than 1 and statistically significant, confirming the benefits of riluzole in preventing patients from HF (Figure S6).

## DISCUSSION

HF is a significant health problem for the United States, in large part because of the aging population. [1, 2] In the United States, approximately 115 million people have hypertension, 100 million have obesity, 92 million have prediabetes, 26 million have diabetes, and 125 million have atherosclerotic CVD [2]. These known risk factors carry a high HF‐development population risk, thereby a large proportion of the US population can be categorized as being at‐risk for HF with aging. Identification of a potential preventative therapy for the development of heart failure is warranted. To this end, this retrospective cohort study of ACC/AHA Stage A or B matched population of ALS patients showed riluzole is associated with a significantly lower rate of HFrEF incidence. The reduction was noted early in exposure and remained consistent throughout the 2‐year follow‐up. Additionally, stratifying the cohort by age showed that riluzole exhibits a reduction in HFrEF incidence primarily in the patients <65 years. A reduction was not found with HFpEF due to an underpowered patient representation. After adjusting for baseline characteristics, riluzole was not associated with a lower HF admission rate, potentially due to the calculated lower HF incidence throughout the study follow‐up.

Riluzole is indicated for the treatment of ALS, so to study the effects of this agent on the HF incidence required using ALS‐diagnosed patients. ALS is a progressive neurodegenerative disease which is the most common form of motor neuron disease. It is most commonly sporadic in presentation usually with no family history [24]. Importantly, recent findings point toward a progressive dysautonomia with potential cardiac involvement [25, 26, 27]. Furthermore, aggregates of phosphorylated TDP‐43, a marker of myogenic degeneration in ALS, might also be found in the cardiac muscle [28]. Study enrollment was based on lack of documented HF in the ALS patients, supporting that the cohort is representative of the large U.S. population at risk for HF, independent of ALS diagonsis [4].

The gradual insidious mechanistic development of heart dysfunction to HFrEF involves cardiac fibrosis, wall stress/stretch, hypertrophy, inflammation, microvascular endothelial dysfunction, or neurohormones [29]. Therapeutic agents effective in preventing HF by affecting one or more of these mechanistic pathways include diuretics, angiotensin‐converting enzyme and angiotensin receptor inhibitors, beta‐adrenergic receptor blockers, mineralocorticoid receptor antagonists, statins, and Na^+^‐glucose co‐transporter 2 (SGLT2) inhibitors [30, 31, 32, 33, 34, 35, 36]. Riluzole may have a therapeutic mechanism distinct or similar from these agents.

The exact mechanistic role of riluzole in reducing HF incidence remains un‐elucidated; however, there are several potential pathways that may be postulated. Riluzole is a neuroprotective agent that biologically quiets glutamate neurotransmission [37, 38]. Riluzole reduces Na^+^ current, in part, through voltage‐gaited brain‐type Na^+^ channel isoforms [16, 20, 21]. Increasing intracellular Na^+^ levels leads to intracellular Ca^2+^ overload through the Na^+^/Ca^2+^ exchange, which in turn impacts cardiac contraction and relaxation [5, 39, 40]. The late Na^+^ current is recognized as a common feature of HF and an attractive target for modulation of cardiac Ca^2+^ handling [9]. Intriguingly, empagliflozin, an Na^+^/glucose cotransporter‐2 inhibitor (SGLT2i) has cardioprotective HF effects by reducing late Na^+^ current and thereby ameliorating spontaneous Ca^2+^ release, which may have detrimental effect on contraction [3, 41, 42, 43]. Furthermore, the SGLT2is trigger a fasting‐like transcriptional nutrient deprivation signaling to lower Na^+^ concentrations in cardiac myocytes, potentially contributing to their cardioprotective effects [44].

The heart through its constant workload has a cardiac energy supply and metabolism which is tightly regulated. This regulation is compromised by the development of HF which leads to an energy starvation state [45]. Mitochondrial Ca^2+^ regulates oxidative phosphorylation including production of adenosine triphosphate (ATP) and reactive oxygen species (ROS) [46]. Elevated intracellular Na^+^ impairs mitochondrial Ca^2+^ accumulation at high pacing rates, leading to decreasing NADH/NAD^+^ redox potential and giving rise to elevated reactive oxygen species formation [47, 48]. Notably, pharmacological enhancement of SK channels in a rat model of pressure overload induced hypertrophy reduced emission of damaging mitochondrial reactive oxygen species, and improved cytosolic Ca^2+^ cycling, thereby reducing wasteful spontaneous sarcoplasmic reticulum Ca^2+^ release [14, 49]. Since riluzole enhances SK channel function [17], the beneficial effect of this medication highlighted in this study may also stem from mitochondrial stabilization achieved through SK channel modulation.

The autophagy pathway is an evolutionary conserved lysosomal degradation which plays a key role in cellular bioenergetics and cytoplasmic quality by removing peroxisomes that are major sources of reactive oxygen species and cellular dysfunction in cardiac myocytes [50, 51]. Riluzole has shown to upregulate autophagy pathways and thereby palliate various types of cancers in vitro [52, 53, 54, 55]. Of note, SGLT2 inhibitors have also been established to promote autophagy in diverse organs, including the heart [56, 57]. Thus, the induction of autophagy by SGLT2 inhibitors as well as riluzole may underlie the cardioprotective effect of these agent in cardiomyopathy. Additionally, riluzole increases the amount of heat shock response through the hsp70 receptor gene to potentially amplify cardioprotective effect [58].

### Limitations

Interpreting the study findings, we should take several limitations into consideration. One of the major concerns was attributed to the lack of mortality information. In the database, patients who died before they developed HF were censored from the follow‐up, which could have an influence on the cumulative incidence. Given that we matched chronic conditions that increase mortality, patients with more ALS symptoms and at a higher chance of case fatality would receive riluzole. This likely led to an overestimation of the cumulative HF incidence in the riluzole group. Therefore, although there is a possibility of differential mortality and differential censoring between the exposure and control groups, the effects of mortality likely underestimated the effect of riluzole and would decrease the chance of making a type I error. Another limitation of our approach is the right censoring incurred due to the gap between the date of ALS onset and riluzole dispensing. According to our analysis, the median time to receive riluzole from the first ALS diagnosis was 2 days, which would not introduce bias in calculating the treatment effect. Lastly, our findings should be considered in light of general limitations in the use of reimbursed claims for the commercially insured population, including limited generalizability, miscoding, and unobserved confounders.

## CONCLUSIONS

This retrospective cohort study of an ACC/AHA Stage A or B matched population of ALS patients showed riluzole is associated with a significantly lower rate of HFrEF incidence. The reduction was noted early and remained throughout the 2‐year follow‐up. These results support further experimental and clinical research into the potential protective role of riluzole in heart failure incidence.

## AUTHOR CONTRIBUTIONS

**Kibum Kim:** Conceptualization; data curation; formal analysis; investigation; methodology; supervision; validation; writing – original draft; writing – review and editing. **Sodam Kim:** Data curation; formal analysis; writing – original draft; writing – review and editing. **Margaret Katana:** Investigation; writing – review and editing. **Dmitry Terentyev:** Conceptualization; funding acquisition. **Przemysław B. Radwański:** Conceptualization; funding acquisition; formal analysis; investigation; methodology; validation; writing – review and editing. **Mark A. Munger:** Conceptualization; methodology; data curation; investigation; validation; formal analysis; supervision; writing – original draft; writing – review and editing.

## FUNDING INFORMATION

The National Institutes of Health supported this work from grants R01HL155378 and R01NS121234 (to Radwański), R01HL142588 and R01HL166604 (to Terentyev).

## CONFLICT OF INTEREST STATEMENT

The authors declare that they have no conflict of interest associated with the content of this manuscript.

## Supporting information


Figure S1.

## Data Availability

The data that support the findings of this study are available from the corresponding author upon reasonable request.
