# Supplementary material for: Riluzole is associated with reduced risk of heart failure
Source: Eur J Neurol. 2025 Jan 9;32(1):e70033. doi: 10.1111/ene.70033 (PMC11716981; doi:10.1111/ene.70033)
Supplement: Supplementary file 1 — Figure S1. [file ENE-32-e70033-s001.docx]

**Supplement Tables & Figures**

Supplement Table 1. ICD-9/ICD10 Codes for ALS Diagnosis, and NDC Codes for Riluzole

|  | Codes |
| --- | --- |
| ICD codes for ALS diagnosis | 335.20, 335.21, and G12.21 |
| NDC codes for riluzole | 24987070060, 42291072360, 42291077560, 60505328506, 62756053818, 62756053883, 62756053886, 62756053888, 64980019106, 67877028605, 67877028610, 67877028614, 67877028660, 67877028690, 68084090825, 68084090895, 68462038110, 68462038160, 69076020060, 70510220102, 70515070060, 70726030301, 70726030302, 00075770060, 00115300013, 00378414591, 24987070060, 42291077560, 60505328506, 62756053886, 64980019106, 67877028660, 68462038160, 70515070060 |

Supplement Table 2. Baseline Characteristic for Analytic Cohorts for HF Admission

|  | **Riluzole**  **(n=4,441)** | **No riluzole ALS**  **(n=6,937)** |  |
| --- | --- | --- | --- |
| **Characteristic** | **N (%)** | **N (%)** | **p-value** |
| **Demographic characteristics** |  |  |  |
| Male | 2456 (55.68) | 4060 (58.53) | <0.01 |
| Age |  |  |  |
| Mean ± SD | 59.08 (12.68) | 62.31 (14.73) | <0.01 |
| Median [IQR] | 60 [52 - 66] | 62 [54 - 73] |  |
| Age category, yrs |  |  | <0.01 |
| <45 | 503 (11.40) | 710 (10.23) |  |
| 45-64 | 2602 (58.99) | 3343 (48.19) |  |
| ≥65 | 1306 (29.61) | 2884 (41.57) |  |
| Region |  |  | <0.01 |
| Northeast | 1033 (23.42) | 1524 (21.97) |  |
| North Central | 1003 (22.74) | 1778 (25.63) |  |
| South | 1588 (36.00) | 2343 (33.78) |  |
| West | 728 (16.50) | 1151 (16.59) |  |
| Unknown | 59 (1.34) | 141 (2.03) |  |
| Medicare | 1245 (28.22) | 2814 (40.57) | <0.01 |
| **Charlson’s Comorbidity Index Score** |  |  |  |
| Mean ± SD | 2.32 (1.80) | 2.95 (2.21) | <0.01 |
| Median [IQR] | 2 [1 - 3] | 3 [1 - 4] | <0.01 |
| **Baseline comorbidity** |  |  |  |
| HIV | 8 (0.18) | 19 (0.27) | 0.32 |
| Acute Myoardial Infarction | 51 (1.16) | 227 (3.27) | <0.01 |
| Cancer | 286 (6.48) | 590 (8.51) | <0.01 |
| Cerebrovascular Accident | 731 (16.57) | 1299 (18.73) | <0.01 |
| Congestive Heart Failure | 87 (1.97) | 522 (7.52) | <0.01 |
| Connective Tissue Disorder | 134 (3.04) | 260 (3.75) | 0.04 |
| Dementia | 38 (0.86) | 175 (2.52) | <0.01 |
| Diabetes | 508 (11.52) | 1164 (16.78) | <0.01 |
| Diabetes w/ Complication | 2 (0.05) | 6 (0.09) | 0.50 |
| Liver disease, mild - moderate | 8 (0.18) | 47 (0.68) | <0.01 |
| Cancer, Metastatic Solid Tumor | 41 (0.93) | 78 (1.12) | 0.32 |
| Paraplegia | 179 (4.06) | 379 (5.46) | <0.01 |
| Peptic Ulcer | 27 (0.61) | 65 (0.94) | 0.06 |
| Peripheral Vascular Disease | 131 (2.97) | 338 (4.87) | <0.01 |
| Pulmonary Disease | 521 (11.81) | 1161 (16.74) | <0.01 |
| Renal Disease | 43 (0.97) | 59 (0.85) | 0.49 |
| Liver Disease, severe | 1 (0.02) | 20 (0.29) | <0.01 |
| **Baseline medication** |  |  |  |
| ACEi | 697 (15.80) | 889 (12.82) | <0.01 |
| ARB | 387 (8.77) | 400 (5.77) | <0.01 |
| Beta Blocker | 815 (18.48) | 1063 (15.32) | <0.01 |
| Calcium Channel Blocker | 596 (13.51) | 741 (10.68) | <0.01 |
| Other CV drugs* | 76 (1.72) | 191 (2.75) | <0.01 |
| Antidiabetic agents, Insulin | 89 (2.02) | 202 (2.91) | <0.01 |
| Antidiabetic agents, Metformin | 149 (3.38) | 160 (2.31) | <0.01 |
| Antidiabetic agents, SGLT2i | 14 (0.32) | 9 (0.13) | 0.03 |
| Antidiabetic agents, other | 138 (3.13) | 215 (3.10) | 0.93 |
| Antiplatelet agents | 186 (4.22) | 271 (3.91) | 0.41 |
| Anti-lipid agent | 1280 (29.02) | 1510 (21.77) | <0.01 |
| Spironolactone | 27 (0.61) | 58 (0.84) | 0.18 |

*Other CV drugs include alpha-beta blockers, antiarrhythmic agents and cardiac glycosides

Supplement Figure 1. Two-year Cumulative Incidence and Hazard Ratio estimates of HF onset for Riluzole: Unmatched Cohort

| Log-rank test, p-value < 0.0001 | | | | | | | | | | | | |  |
| --- | --- | --- | --- | --- | --- | --- | --- | --- | --- | --- | --- | --- | --- |
|  | | | | | | | | | | | | |  |
| Month after index date | | | | | | | | | | | | | |
| Number at Risk | | | | | | | | | | | | | |
| Riluzole | 2161 | | 1177 | 783 | | 568 | 421 | | 344 | 281 | | 238 | |
| ALS Control | 3329 | | 1938 | 1367 | | 1059 | 838 | | 686 | 567 | | 469 | |
| Hazard Ratio [95 Confidence Interval] | | | | | | | | | | | | |  |
|  | | 3 months | | | 6 months | | | 1 year | | | 2 years | |  |
| HR_crude_ | | 0.398 [0.271 - 0.585] | | | 0.361 [0.256 - 0.510] | | | 0.373 [0.271 - 0.513] | | | 0.399 [0.297 - 0.537] | |  |
| HR_adjusted_ | | 0.476 [0.323 - 0.703] | | | 0.428 [0.302 - 0.607] | | | 0.448 [0.324 - 0.619] | | | 0.466 [0.345 - 0.630] | | |

Supplement Figure 2. Two-year Cumulative Incidence and Hazard Ratio estimates of HF admission for Riluzole: Unmatched Cohort, exploratory cohort with or without baseline HF.

| Log-rank test, p-value = 0.0429 | | | | | | | | | | | | | | |
| --- | --- | --- | --- | --- | --- | --- | --- | --- | --- | --- | --- | --- | --- | --- |
|  | | | | | | | | | | | | | | |
| Month after index date | | | | | | | | | | | | | | |
| Number at Risk | | | | | | | | | | | | | | |
| Riluzole | 2220 | | 1199 | 801 | | 584 | 434 | | 358 | 297 | | 255 |  |  |
| ALS Control | 3626 | | 2111 | 1493 | | 1154 | 928 | | 767 | 642 | | 532 |  |  |
| Hazard Ratio [95 Confidence Interval] | | | | | | | | | | | | | | |
|  | | | 3 months | | | 6 months | | | 1 year | | | 2 years | | |
| HR_crude_ | | | N/A | | | 0.305 [0.117 - 0.791] | | | 0.281 [0.118 - 0.668] | | | 0.248 [0.105 - 0.583] | | |
| HR_adjusted_ | | | N/A | | | 0.412 [0.135 - 1.261] | | | 0.477 [0.175 - 1.297] | | | 0.411 [0.154 - 1.096] | | |

Supplement Figure 3. Two-year Cumulative Incidence and Hazard Ratio estimates by HF subtype (HFpEF vs. HFrEF): Unmatched Cohort Analysis

| 1. **HFpEF onset, unmatched cohort**   Log-rank test, p-value = 0.2050 | | | | | | | | | | | | | | 1. **HFrEF onset, unmatched cohort**   Log-rank test, p-value = 0.0026 | | | | | | | | | | | | | |
| --- | --- | --- | --- | --- | --- | --- | --- | --- | --- | --- | --- | --- | --- | --- | --- | --- | --- | --- | --- | --- | --- | --- | --- | --- | --- | --- | --- |
|  | | | | | | | | | | | | | |  | | | | | | | | | | | | | |
| Month after index date | | | | | | | | | | | | | | Month after index date | | | | | | | | | | | | | |
| Number at Risk | | | | | | | | | | | | | | Number at Risk | | | | | | | | | | | | | |
| Riluzole | 2176 | | 1188 | 791 | | 576 | 428 | | 352 | 290 | | 247 | | Riluzole | 2179 | | 1191 | 792 | | 575 | 428 | | 352 | 290 | | 248 | |
| ALS Control | 3386 | | 1998 | 1419 | | 1102 | 885 | | 727 | 604 | | 499 | | ALS Control | 3389 | | 1997 | 1421 | | 1104 | 890 | | 726 | 604 | | 497 | |
| Hazard Ratio [95 Confidence Interval] | | | | | | | | | | | | | | Hazard Ratio [95 Confidence Interval] | | | | | | | | | | | | | |
|  | | | 3 months | | | 6 months | | | 1 year | | | 2 years | |  | | | 3 months | | | 6 months | | | 1 year | | | 2 years | |
| HR_crude_ [95CI] | | | 0.77  [0.34 - 1.71] | | | 0.68  [0.34 - 1.39] | | | 0.61  [0.32 - 1.19] | | | 0.67  [0.36 - 1.25] | |  | | | 0.51  [0.20 - 1.28] | | | 0.31  [0.13 - 0.75] | | | 0.36  [0.17 - 0.79] | | | 0.35  [0.17 - 0.71] | |
| HR_adjusted_ [95CI] | | | 0.99  [0.44 - 2.23] | | | 0.78  [0.38 - 1.60] | | | 0.68  [0.34 - 1.33] | | | 0.77  [0.41 - 1.45] | |  | | | 0.55  [0.22 - 1.40] | | | 0.33  [0.14 - 0.80] | | | 0.39  [0.18 - 0.85] | | | 0.39  [0.19 - 0.80] | |

Supplement Figure 4. Two-year Cumulative Incidence and Hazard Ratio estimates of HF onset. Patients with baseline ALS diagnosis only.

| Log-rank test, p-value <0.0001 | | | | | | | | | | | | | |
| --- | --- | --- | --- | --- | --- | --- | --- | --- | --- | --- | --- | --- | --- |
|  | | | | | | | | | | | | | |
| Months after index date | | | | | | | | | | | | | |
| Number at Risk | | | | | | | | | | | | | |
| Riluzole | 1781 | | 935 | 590 | | 415 | 293 | | 237 | 185 | | 153 |  |
| ALS Control | 3329 | | 1938 | 1367 | | 1059 | 838 | | 686 | 567 | | 469 |  |
| Hazard Ratio [95 Confidence Interval] | | | | | | | | | | | | | |
|  | | | 3 months | | | 6 months | | | 1 year | | | 2 years | |
| HR_crude_ | | | 0.393 [0.259 - 0.595] | | | 0.350 [0.240 - 0.511] | | | 0.379 [0.269 - 0.534] | | | 0.406 [0.294 - 0.560] | |
| HR_adjusted_ | | | 0.454 [0.298 - 0.691] | | | 0.397 [0.271 - 0.581] | | | 0.440 [0.311 - 0.623] | | | 0.458 [0.331 - 0.633] | |

Supplement Figure 5. Two-year Cumulative Incidence and Hazard Ratio of HF estimates by Age group (≥65 years vs. <65 years), Matched Cohort Analysis

| 1. **Age ≥65 years**   Log-rank test, p-value = 0.3034 | | | | | | | | | | | | | | 1. **Age < 65 years**   Log-rank test, p-value < 0.0001 | | | | | | | | | | | | | |
| --- | --- | --- | --- | --- | --- | --- | --- | --- | --- | --- | --- | --- | --- | --- | --- | --- | --- | --- | --- | --- | --- | --- | --- | --- | --- | --- | --- |
|  | | | | | | | | | | | | | |  | | | | | | | | | | | | | |
| Month after index date | | | | | | | | | | | | | | Month after index date | | | | | | | | | | | | | |
| Number at Risk | | | | | | | | | | | | | | Number at Risk | | | | | | | | | | | | | |
| Riluzole | 560 | | 274 | 170 | | 126 | 88 | | 66 | 55 | | 48 | | Riluzole | 1470 | | 838 | 575 | | 418 | 312 | | 263 | 212 | | 176 | |
| ALS Control | 610 | | 344 | 244 | | 187 | 153 | | 127 | 110 | | 90 | | ALS Control | 1527 | | 902 | 634 | | 483 | 381 | | 317 | 257 | | 207 | |
| Hazard Ratio [95 Confidence Interval] | | | | | | | | | | | | | | Hazard Ratio [95 Confidence Interval] | | | | | | | | | | | | | |
|  | | | 3 months | | | 6 months | | | 1 year | | | 2 years | |  | | | 3 months | | | 6 months | | | 1 year | | | 2 years | |
| HR_matched_ [95CI] | | | 0.79  [0.45 - 1.38] | | | 0.61  [0.37 - 1.00] | | | 0.71  [0.45 - 1.12] | | | 0.79  [0.51 - 1.23] | |  | | | 0.40  [0.20 - 0.80] | | | 0.41  [0.23 - 0.74] | | | 0.36  [0.21 - 0.63] | | | 0.37  [0.23 - 0.62] | |
| HR_matched&adjusted_ [95CI] | | | 0.76  [0.44 - 1.32] | | | 0.59  [0.36 - 0.97] | | | 0.70  [0.44 - 1.11] | | | 0.79  [0.51 - 1.22] | |  | | | 0.39  [0.20 - 0.79] | | | 0.40  [0.22 - 0.71] | | | 0.35  [0.20 - 0.61] | | | 0.37  [0.22 - 0.60] | |

Supplement Figure 6. Sensitivity Analysis: Excluding patients on angiotensin converting enzyme inhibitors or angiotensin receptor blockers

|  |  | Hazard Ratio and 95% Confidence Interval |
| --- | --- | --- |
| Matched cohort |  |  |
|  | 90 days |  |
|  | 180 days |  |
|  | 365 days |  |
|  | 730 days |  |
| Unmatched Cohort | 90 days |  |
|  | 180 days |  |
|  | 365 days |  |
|  | 730 days |  |
|  |  |  |

Footnote: Hazard ratios and 95% confidence intervals were calculated from multivariable Cox proportional hazard regression model.

Legend: **Black, Results from cohort including patients on angiotensin converting enzyme inhibitors or angiotensin receptor blockers;** **Gray, Results after excluding patients on angiotensin converting enzyme inhibitors or angiotensin receptor blockers**
